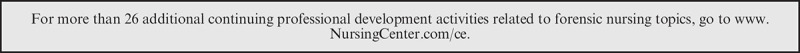# Sexual Violence in Virtual Reality: A Scoping Review

**DOI:** 10.1097/JFN.0000000000000466

**Published:** 2023-12-13

**Authors:** Carolyn M. Porta, Ellen A. Frerich, Sarah Hoffman, Sara Bauer, Vedushi M. Jain, Cynthia Bradley

**Affiliations:** **Author Affiliations:** 1 School of Nursing; 2 Office of Academic Clinical Affairs; 3 School of Public Health, University of Minnesota.

**Keywords:** Adolescents, college age, gaming, harassment, sexual violence, virtual reality, young adults

## Abstract

**Background:**

One in four Americans report experiencing harassment online via social media and interactive gaming, which includes physical threats, stalking, sexual harassment, and sustained harassment.

**Objective:**

The aim of this study was to gain understanding of the state of the science surrounding young adults and sexual violence/harassment harms in virtual reality (VR) as well as possible uses of VR to heal and intervene.

**Methods:**

A scoping review was conducted in early 2023 using the Ovid Synthesis Clinical Evidence Manager and the MEDLINE database. Forty-seven articles met inclusion criteria.

**Results:**

Our review found a growing body of evidence exploring incidents, effects, possible predictors, and initial strategies to prevent sexual violence in VR and to use the modality to positively intervene. Limited research addresses the effects of harms incurred in VR on (re)traumatization of survivors as well as the development and testing of VR tools used to educate, deliver bystander interventions, transform biases and perceptions via embodiment, and promote healing among survivors.

**Conclusion:**

Research addressing sexual violence in VR is needed and should build on the existing peripheral science on gaming and social media environments. Forensic nursing is well positioned to advance strategies of health and safety in VR, just as in the physical world. Incorporating forensic nursing avatars in VR and deploying diverse resources targeted for college-age young adults to prevent harms in VR should be explored safely and ethically. Forensic nurses are also positioned to assess for VR-related harms among patients and to work with private and government sectors to influence regulations and policies.

There are 171 million people worldwide using virtual reality (VR) gaming and tools, and this number is expected to increase as the technology gains popularity in health care, education, workplaces, gaming, and social venues (Blagojević, n.d.). The largest group of video game players is 18–34 years old (36%; (Statista, 2022); many of these engage in VR as part of their gaming experiences. VR is a broad terminology that includes screen-based virtual play and immersive VR play with head-mounted displays and handheld haptics. With the 2023 launch of a new competitive Apple VR visual product, Vision Pro (Apple, 2023), alongside ongoing technological advancements in VR tools (i.e., 10+ VR headsets; Greenwald & Minor, 2023), growth and intensification in VR engagement is inevitable across the United States and the globe.

Although most users report overall positive experiences gaming and engaging in VR, in 2020, 81% also reported they have experienced at least one form of harassment (Anti-Defamation League, 2019). Successful and popular female gamers using a pseudonym identity have reported being stalked, doxxed (i.e., someone has searched out and publicized their real identity, contact information, or other demographics), and, in numerous other ways, sexually harassed and harmed (Anti-Defamation League, 2019; Lorenz & Browning, 2020). Using a crowdsourced tracking sheet in 2018, there were over 400 reports of sexual harassment associated with online and live stream gaming, with specific perpetrator gamer names documented (Anti-Defamation League, 2019). Nearly half of women engaged in social VR spaces report they have experienced at least one occurrence of sexual harassment; men more often report incidents of racist or homophobic comments, or violent threats (McLean & Griffiths, 2018; Oultaw, 2021).

Although national data from 2021 indicate that most respondents endorse experiencing harassment and related harms in the context of social media (e.g., Facebook, Instagram, myriad dating/meet-up apps), increased adolescent and young adult engagement in gaming and VR immersive environments is likely to contribute to these numbers only growing with time. A 2021 Pew survey found that 75% of targets of online abuse—equating to nearly a third of all Americans—say their most recent harassing experience was while using a social media platform (Pew Research Center, 2021). These are important contextualizing data in part because similar patterns of perpetration are occurring in gaming and VR but, for a variety of reasons, might be underreported and underrecognized by those experiencing the harms.

Consider the following additional data about online harassment, including social media and gaming, and the concerning trends by age, gender, and sexual identity. A growing number of Americans reported experiencing more severe forms of harassment, which encompasses physical threats, stalking, sexual harassment, and sustained harassment, whereas only 15% reported experiencing these harms in 2014 (vs. 18% in 2017 and 25% today; Pew Research Center, 2021). Online harassment is a particularly common feature of online life for younger adults, and they are especially prone to facing harassing behaviors in part because of the extent to which their online presence is integrated within their day-to-day living and in part because of their life stage, which is often characterized by having high levels of socialization, seeking out new people and new adventures, establishing new circles of friends as young adults, and exploring new romantic or sexual relationships. Roughly two thirds of adults under 30 years old (64%) reported that they have experienced any form of online harassment activities—making this the only age group in which most have been subjected to these behaviors (Pew Research Center, 2021).

Online harassment appears to be experienced differently among various demographics in type and frequency. Men are somewhat more likely than women to say they have experienced any form of harassment online (43% vs. 38%), although similar numbers of men and women report having faced more severe forms of this abuse (Pew Research Center, 2021). Women, on the other hand, are more likely than men to report having been sexually harassed online (16% vs. 5%) or stalked (13% vs. 9%). Young women are particularly likely to have experienced sexual harassment online (Pew Research Center, 2021). One in three (33%) women under 35 years old report they have been sexually harassed online (vs. 11% of men under 35 years old; Pew Research Center, 2021). Lesbian, gay, or bisexual adults are even more likely to report they have faced harassment online. Roughly seven in 10 report they have encountered any harassment online, and over half (51%) report they have been targeted for more severe forms of online abuse (compared with four in 10 and 23% of adults who identify as heterosexual, respectively). Although these harms may occur anywhere online, social media is the most common venue cited as the source of harassment, with gaming and VR environments comprising a small but growing proportion of reported harassment Pew Research Center (2021).

The relatively lower rates of reported harassment in gaming environments as compared with social media and other online platforms make sense as one considers that men are and have been the predominant users of gaming and, subsequently, of VR. Consequently, and because general harassing behavior within the game or the online chatting accompanying the game has been a cultural norm, it might not be viewed or experienced by most men as harassment or might be viewed as normal and simply part of the gaming experience (e.g., we all do it to each other; it is just what you do in gaming). The virtual nature of online gaming, separated from your competitors or teammates physically yet connected within the game, facilitates escapism and embodiment of a character willing or free to engage in behaviors and words that one would never do in the physical real world. Wolfendale (2007) describes that gaming participants as avatars in their online gaming world “can use their online personas—avatars—to chat, fight, make friends, have sex, kill monsters, and even get married…can also use their avatars to stalk, kill, sexually assault, steal from and torture each other” (p. 111).

Consider this description of the typical modern-day online gaming environment (with or without VR), the visual narratives of some games represent women, men, animals, and deprived social groups in exaggerated and stereotypical ways, encouraging ideals such as machismo—violent male and sexually willing, timid women. Glamorization of sexual violence and representing everything including murder, rape, and other forms of violence in a comic or ludicrous way are not uncommon in games. There are games that perpetuate gender stereotypes. Yet, some other games have the formula of subversion of gender identity embedded in them because of the anonymity permitted in the domain of most games (Rajagopal & Rao, 2016, p. 282).

As more women join the gaming and VR communities, they report experiencing intense harassing behaviors and notably high rates of sexual harassment. Jenson and De Castell (2021) poignantly describe the gaming realm as one composed of “...particularly hostile environments to those identifying or identified as women” (p. 195). In these immersive gaming environments, it is common for young male gamers to take on female avatars in their games; this is not met with similar rates and types of harassing behaviors, likely in part because the young men are known to their gaming peers before taking on a female-appearing avatar and, thus, are treated as male despite the female avatar representation. It is also common for gamers to switch their avatar choices frequently, which possibly lends fellow gamers to harass in more general ways, if they are going to harass, rather than choosing to be sexually explicit and violent.

VR environments pose additional risks. Women have reported experiencing their avatar being cornered or trapped in a virtual room, by one or numerous assailants, who might restrain the avatar or perform other violent or sexual assault behaviors against the avatar. For some gamers, they experience visceral responses to these assaults in their physical real body (e.g., Leonard, 2019; Weiderhold, 2022) and experience retraumatization of previous sexual assault or abuse they experienced in the physical world. As Weiderhold poignantly states in a published interview, “If you've had this [sexual violence] happen to you in the metaverse, it doesn't end when you take off the headset” (Singh, 2022).

We cannot consider harassment and sexual violence experienced in VR without briefly addressing the longevity of sexual violence and its related precursors since video games originated, including an original 1982 video game, Atari's Custer's Revenge, which rewarded level mastery with the soldier character pushing an erect penis against a Native American woman tied to a post (“Custer's Revenge,” 2023). The evolutionary progression of the gaming realm is characterized by dominance of programmers, developers, marketers, and manufacturers who have persisted in advancing sexualization of gaming avatars and characters and embedded sexualization and sexual violence within their gaming scenarios and rewards. More recently, in 2019, a developer (Desk Plant) released Rape Day, a game centered on a serial killer and rapist who, during a zombie apocalypse, rapes and kills women; the game was canceled in 2019 before being publicly released. The developer went on to remove explicit child rape from the game and to sell it via his personal website (Games4Win, n.d.). Complex sociocultural and economic factors further contribute to the prevailing popularity of these games, their stories, and their accompanying visualizations. Beyond the scope of this article, for additional examination of the complicated history, present day, ethics, legalities, and conflicts surrounding video gaming, the metaverse, and sexual violence, see Hoffin and Lee-Treweek (2020), Jenson and De Castell (2021), Ramirez et al. (2023), and Wiederhold (2022).

VR technology potentially exacerbates fundamental harms inherent in gaming because of the consuming, immersive, and embodiment nature of VR. These exacerbated harms include not only the sexual violence occurring in games but also the sexual violence that is experienced quite powerfully vis-à-vis the avatar the gamer has chosen to embody in these multidimensional virtual worlds, where “sound effects and three-dimensional graphics generate hyperreal excitement from the virtual act which perhaps excels the feel of committing murder, rape, and suicide or the thrill of hunting” (Rajagopal & Rao, 2016).

Recognizing the sexual harassment and violence occurring in VR environments, legal scholars have recently engaged in discourse regarding whether or not a human person ought to be held accountable for the actions of their nonhuman representation (i.e., avatar) in VR spaces (Barfield et al., 2020; Pew Research Center, 2021). Philosophical scholars have further explored the extent to which virtual rape in a virtual world can be viewed as rape in our physical and legal world, concluding the most serious legal claim could be “harassment” (see Strikwerda, 2015). Although these deliberations and debates have important implications for policy and regulatory actions specific to gaming environments and parameters, as well as to the criminal or legal consequences of these behaviors for offenders and victims, it is not likely to be resolved in the near term. Meanwhile, violence in gaming persists, and sexual violence in VR continues to yield serious harms and negative outcomes for victims and, potentially, witnesses to the violence. Notably, gaming experiences of stalking, rape, and child abuse are, for many current and future gamers, representative of what they have experienced in their past in the physical world; encountering these in a game has potential to result in new trauma, retraumatization, and realized threats to mental and physical well-being (Chivers-Wilson, 2006; Rajagopal & Rao, 2016).

To assess the state of the science and knowledge and understanding regarding sexual violence in VR gaming environments, we conducted this scoping review.

## Methods

The Arksey and O'Malley (2005) methodology for conducting scoping studies was used. In late spring/early summer 2023, a search was conducted in Scopus using the following search strategy: TITLE-ABS-KEY (((groping OR groped) OR (sexual* W/2 (assault* OR harass* OR violence))) AND TITLE-ABS-KEY (“virtual reality” OR vr OR metaverse OR “augmented reality” OR “video gam*” OR gaming OR “online gam*” OR xr OR “extended reality” OR “mixed reality”). All identified records were imported into Rayyan, a web-based program designed to facilitate systematic review processes (Rayyan Systems, 2022).

Inclusion criteria for advancing to full-text publication review were

- scoping, systematic, and literature reviews on sexual violence in VR environments, including documenting prevalence and incidence;
- research describing sexual violence and related negative experiences in VR environments; and
- research developing or testing VR or gaming interventions addressing factors associated with sexual violence.

Exclusion criteria included

- research not written or translated in English;
- research published before 2013; and
- research focused on sexual violence and technology beyond VR and gaming (i.e., dating apps, sexting, revenge porn).

The principal investigator (C.M.P.) and graduate assistants independently assessed abstracts and the full text, as needed, to determine whether or not inclusion criteria were met. In the case of disagreement, the two reviewers discussed initial reasoning and resolved any disagreements with the senior researcher. Reviewers read the full text of selected studies, subsequently sorted results by type of study (review, descriptive research, and intervention research), and then created thematic subcategories to facilitate synthesis and identifying granular implications for forensic nursing practice, research, and policy efforts.

Numerous relevant terms were identified throughout this scoping review process and are summarized in Supplemental Digital Content 1 (http://links.lww.com/JFN/A131) to aid the readers not as familiar with this topical area.

## Results

**FIGURE 1:**
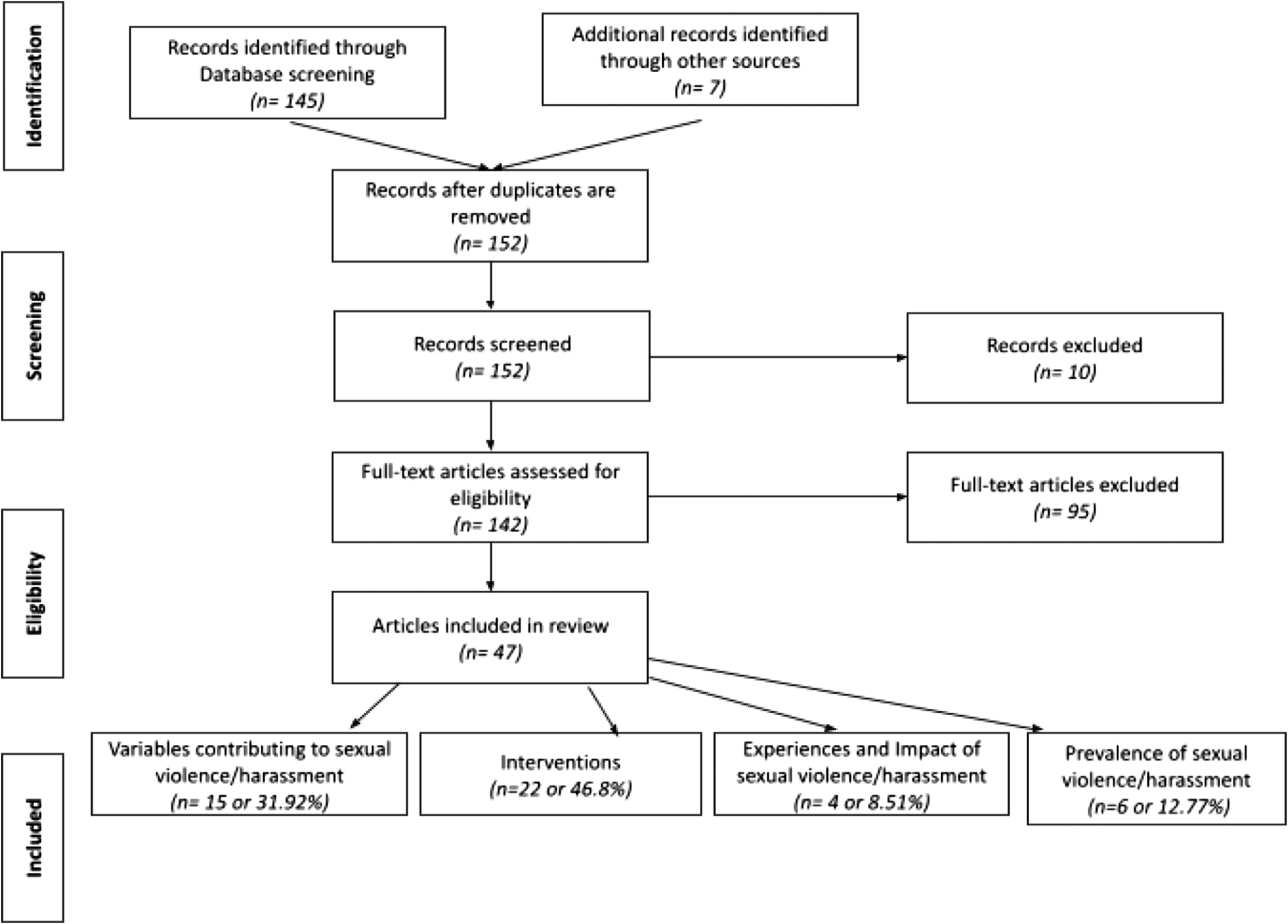
Preferred Reporting Items for Systematic Reviews and Meta-Analyses (PRISMA).

The database search identified 145 potential nonduplicative articles; seven additional sources were identified through additional citation analysis. Ninety-five articles were excluded for not meeting inclusion criteria. Forty-seven records were subsequently included in this review (see Figure 1).

### Study Characteristics

Of the 47 articles, five were reviews; 31 were research studies characterizing sexual violence prevalence, predictors, experiences, and relevant non-VR gaming interventions; and 11 were studies specifically testing VR as an intervention strategy. Most of the studies recruited college-age young adults, or adults, and were conducted in college environments or within the community. Numerous countries were represented in the reviews, but much of the original research was conducted in North America.

### Reviews

Five relevant reviews were identified, each offering unique and important direct and peripheral contributions to the state of the science surrounding sexual violence and harassment in VR and broader gaming environments (Henry & Powell, 2018; Moor & Anderson, 2019; Park & Kim, 2023; Patel & Roesch, 2022; Xue et al., 2021). These are briefly summarized, from oldest to newest. Henry and Powell (2018) conducted a review of technology-facilitated sexual violence specific to adult populations, recognizing that much of the prior research focus has historically been on children and adolescents. Key findings of their review show very limited knowledge about the prevalence and impacts of sexual violence as well as inconsistent and complicated working definitions of numerous terms and concepts scientists are attempting to measure and examine with respect to online/digital/virtual spaces and sexual violence. They note the growing concerns and need for studies in this space, recognizing that “digital technologies then serve as a tool to perpetrate more conventional forms of gender- and sexuality-based violence and harassment, with different effects and impacts due to anonymity, the failure of regulation, as well as the sheer speed and vast reach of the Internet” (p. 204).

Moor and Anderson (2019) conducted a review of the literature specific to dark personality traits and online antisocial behaviors. Examining 24 studies published between 2014 and 2019, they found compelling evidence for a correlational relationship between technology-facilitated sexual violence, specifically revenge porn, and the dark tetrad of personality traits (i.e., sadism, narcissism, psychopathy, and Machiavellianism) and found that psychopathy predicted not only revenge porn behaviors but also additional concerning online behaviors that could be present in VR, including cyberbullying, cyber-aggression, and cyberstalking.

Xue et al. (2021) conducted a scoping review specific to using VR or augmented reality to study bystander behaviors surrounding interpersonal violence. Eleven studies met their inclusion criteria, and of these, only two actually used VR to deliver a bystander intervention to prevent bullying among children. Their review yielded no studies using VR to promote bystander interventions specific to violence.

Park and Kim (2023) took a different approach to evaluate effectiveness of bystander interventions targeting interpersonal violence and sexual assault, by identifying studies aiming to achieve behavioral outcomes irrespective of the mode of intervention delivery. Thirty-five studies were reviewed, and 135 outcome measures were categorized into eight domain outcomes and compared across studies. Most studies involved high school or college-age students, with intervention deliveries including offline, online, web-based, and one VR intervention (included in our review as well). Results varied by country of origin, with plausible explanations for varied results provided by the authors.

Finally, Patel and Roesch (2022) conducted a recent meta-analysis of findings specific to sexual violence summarized in 19 studies and involving 32,247 adult participants. Although focused primarily on aspects of revenge porn (sharing images, sexting), the review provides important prevalence data for victimization (ranging from 7.2% to 17.6%) and perpetration (ranging from 2.7% to 12%) of sexual violence behaviors. Furthermore, they conducted a qualitative meta-analysis of nine studies (*n* = 3,990 participants) of sexual violence correlated with numerous unsurprising mental health effects including anxiety, depression, posttraumatic stress disorder (PTSD), suicidality, self-esteem, and self-harm. Diverse definitions of sexual violence including varying definitions of perpetration and victimization continue to negatively impact the science, and the authors call for consensus around definitions. Furthermore, with most studies occurring in the West and with college students, there is a recognized need for greater diversity in study environments, recruitment efforts, and tracking of participant demographics.

### Studies Examining Sexual Violence and Related Characteristics in VR and Gaming Environments, and Intervening to Promote Health and Prevent Harms

Thirty-one articles described findings from numerous studies describing the scope and characteristics of sexual violence in VR and gaming environments, the experiences of those who have been harassed in gaming, and the outcomes of efforts to intervene on many targeted behaviors and attitudes (e.g., bystander intervention, empathy). Refer to Supplemental Digital Content 2 (http://links.lww.com/JFN/A132). We intentionally included a few studies that focused on the sexual harms within online gaming that might not have been explicitly VR because it is clear that many of these live interactive online gaming two-dimensional environments not only contain sexual harassment and violence threats but also are poised to adapt to three-dimensional VR games. The limited research that is specific to VR immersive environments necessitates attention to studies focused on proximal gaming precursors and longer-standing gaming environments.

Of note, 10 of the articles represent studies of four research teams that have successfully advanced their work from pilot studies to larger scale experimental trials with college students, namely, Research Team 1: Burnay et al. (2019) and Noël et al. (2021); Research Team 2: bib26Fox and Tang (2014, 2016), Tang and Fox (2016), and Tang et al. (2020); Research Team 3: Oliveira and Fonseca (2019) and Fonseca et al. (2018); and Research Team 4: Potter et al. (2019) and Potter et al. (2021).

#### Prevalence Studies of Sexual Violence and Harassment Experienced Online, in Gaming, and in VR

Four studies explicitly sought to understand the prevalence of sexual harms in these online gaming environments; sample sizes ranged from 127 to 1,682. The largest cross-sectional study involved 14- to 18-year-olds; the other studies involved respondents between 14 and 74 years old. Rates of sexual harassment ranged from 34.2% to 84.3%, with one study showing women received 11 times more sexual harassment comments than their male gaming counterparts. Study measurements and variable definitions likely influence some differences observed in results (see Supplemental Digital Content 2, http://links.lww.com/JFN/A132).

#### Studies Identifying Predictors and Characteristics Associated With Sexual Violence and Harassment Online, in Gaming, and in VR

Twelve studies provide insights into the characteristics of the harms and the offenders, with many research teams working to identify the predictors of online sexual violence and harassment. Most of the 12 utilized cross-sectional survey methods with young people, with some only with male participants (Jagayat & Choma, 2021; Seo et al., 2022; Tang & Fox, 2016). There were four unique studies that employed case study, discourse analysis, content analysis, and phenomenological designs (Alexander, 2015; Cheong et al., 2015; Cote, 2017; Mercer & Parkinson, 2014; see Supplemental Digital Content 2, http://links.lww.com/JFN/A132). Endorsement of masculine norms and social dominance, greater length of time engaged in specific games (e.g., exposure to hostility), and offline sexist beliefs were found predictive of likelihood to engage in sexual harms online in gaming; length of time and frequency of gaming were also associated with increased potential for victimization. Amid general consistency in findings and trends, one study (Ferguson & Colwell, 2020) documented no correlation between the participant's exposure to sexualized content in video games and their sexist attitudes or empathy toward a victim of rape. The authors provided no explanation for this unexpected finding; however, in moderation analysis, they did observe that, among those with higher scores on the trait aggression measure, there was an association between sexualized content and lower sexist belief scores, implying that some inherent or static personal characteristics affect some responses to some exposures.

#### Studies on Impact of Sexual Violence and Harassment Experienced Online, in Gaming, and in VR

Four studies document the impacts of online harassment, broadly, and in gaming/VR (Fox & Tang, 2016; Michikyan et al., 2014; Salerno-Ferraro et al., 2022; Sobieraj, 2018). Cross-sectional and qualitative, these studies gained insights from women (one of the four involved male and transgender victims) regarding their experiences, including the types of harassment they experienced in varied online environments, how they coped with those experiences (healthy and unhealthy ways), and the lasting effects of the experiences. Sobieraj (2018) included analysis and categorization of three broad strategies offenders use to harass online, which are consistent with harassing in the physical/real world, namely, intimidating, shaming, and discrediting.

#### Use of Video Games to Educate, Promote Bystander Intervention, Reduce Rape Myths, and Promote Empathy

Numerous researchers are capitalizing on the appeal of gaming for young adults and testing outcomes of interventions developed via various video gaming strategies. For example, Jozkowski and Ekbia (2015) used a participatory approach to develop and test a video game called College Craft designed to educate college students about rape culture and sexual consent. One hundred forty-one college students engaged with the game and provided a broad array of feedback about the game as well as provided premeasures and postmeasures of content-relevant knowledge. The authors reported a mixed response from the college students, as well as a general demonstration of knowledge gained through the gaming experience, and go on to conclude that gaming could be a viable sexual violence prevention educational tool that warrants further inquiry. Similarly, Potter et al. (2019) described a co-creation process with college students to develop two distinct video games (i.e., trivia, an adventure) designed to positively influence attitudes toward bystander interventions.

Numerous studies were found reporting results from randomized controlled trials testing video gaming and video game interventions to determine impacts on various outcomes including attitudes (e.g., sexism), beliefs (e.g., rape myths, stereotypes, efficacy), and behaviors (bystander intervention, harassment behaviors, victim blaming, and sexist jokes; see Supplemental Digital Content 2, http://links.lww.com/JFN/A132). Most studies show results in the expected directions; for example, Potter et al. (2021) describe the results of a three-arm randomized controlled trial involving 227 undergraduate college students; all three conditions showed immediate increases in scores of bystander efficacy and intention, with only women in the adventure game condition sustaining bystander efficacy improvements at follow-up. The authors concluded that video game interventions should be cautiously considered alongside traditional bystander interventions with college students but not necessarily replace the traditional modalities of delivering education, harm reduction, and health promotion tools.

In another example, Burnay et al. (2019) examined whether the content of video games could serve as a predictor of whether or not a young person would engage in either offline sexual violence or sexual violence in the online space. When controlling for individual personality variables outlined above, Burnay et al. predicted that playing a video game with sexualized female characters would increase sexual harassment among participants (both cisgender men and cisgender women). Results supported the hypothesis, with the researchers concluding that “sexual harassment levels toward a female partner were higher for participants who played the game with sexualized female characters than for participants who played the same game with non-sexualized female characters” (p. 214). In addition, their findings suggest “that sexualization of female characters in a video game can be a sufficient condition to provoke online sexual harassment toward women [in the metaverse]” (Burnay et al., 2019, p. 214).

### Using VR Environments to Intervene

Eleven studies report results from experimentally testing VR immersive environments and gaming technologies to intervene toward specific outcomes relevant to the prevention of and response toward sexual violence; these were logically and naturally organized into four thematic groups of studies, which are described below.

#### VR Environments Delivering Bystander Interventions

Only one article was found describing the development and testing of a VR bystander intervention to prevent sexual violence (Jouriles et al., 2016). Jouriles et al. described testing a VR bystander intervention simulation experience with 91 college students to assess the value of this novel tool in measuring their bystander behaviors. In the study, students participated in seven VR simulation scenarios, four of which posed a risk of unwanted sexual contact; the participant was able to act to try to prevent harm in any of the scenarios. Typical bystander intervention measures were collected. Study results showed positive correlations between the bystander behaviors in the simulation and students' sense of responsibility, efficacy and intention to intervene, and self-reported bystander behaviors. It is possible this type of immersive tool could be used as a proxy for actual bystander intervention behaviors and related outcomes, especially if safety and ethical considerations continue to inform the immersive experience.

#### VR Environments to Deliver Educational Interventions, Not Explicitly Targeting Bystander Outcomes

Only one article provides insights into the development and testing of VR gaming interventions to intervene on participant perspectives, attitudes, and intentions, but not specifically addressing bystander constructs (Sadeh-Sharvit et al., 2021). Sadeh-Sharvit et al. (2021) described the development of a VR simulation centered on an interview process in which the interviewee is sexually harassed; piloted with five young adult women, the VR scenario was designed to strengthen women's response to incidents like this in the physical world when they occur. The authors note that key findings included not only the female participants' sense of fear and inability to respond but also that some of these women had previous experiences come to mind (resurface) for them, which is a critical finding as one considers appropriate and safe use of VR immersive scenarios involving harassment. Despite these concerning results, the authors concluded that VR remains a viable and important tool to equip women with “effective responses” to sexual harassment.

#### Testing the Effects of Embodiment in VR to Influence Biases and Empathy

Six articles (including a published conference abstract) describe five studies that used VR to facilitate various aspects of embodiment and test hypotheses that doing so will positively influence gender biases, empathy, and beliefs surrounding sexual violence, harassment, and vulnerability of women (Al-Zubeidi et al., 2021; Neyret et al., 2020; Steinfeld, 2019; bib78Ventura et al., 2021, 2022; Wu & Chen, 2022). Al-Zubeidi et al. (2021) report their development of A Walk Alone, a VR immersive experience in which the individual experiences what it feels like to walk alone as a woman in the evening; results from any pilot work are not reported, and it is unclear if the product has been tested or researched in any depth. Neyret et al. (2020) recruited 60 men into one of three conditions: embodiment as a woman experiencing sexual harassment in a bar with men, embodiment as one of the harassing men, and a control condition consisting of an empty bar. This VR experience was followed a week later by a VR exercise in which the men were instructed to deliver shocks to a woman; those who had been embodied as a woman in the original experiment delivered half as many shocks as the other experimental condition, with the control group in the middle. Embodiment of a woman was not found to be entirely helpful as the authors had hypothesized. Of note, the authors also found they had to reexamine the ethical aspects and potential threats that arose in using this study protocol, which is useful to future researchers considering similar methodologies. Steinfeld (2019) explored the extent to which a VR immersive experience differed in impact from a narrative or two-dimensional intervention; using a scenario of ongoing verbal sexual harassment of an employee by their manager, the three arms were compared on changes in attitudes and empathy toward the victim. The VR immersive condition appeared to have the greatest impact, particularly in positively influencing stereotypical views of men regarding sexual harassment. bib78Ventura et al. (2021; 2022) describe development and testing of a VR immersive experience intended to influence empathy by having 44 men embody the woman being harassed in the VR scenario. Tested against a simple narrative about the harassment, the VR experience yielded stronger positive outcomes in increasing empathy, a sense of oneness with the female victim, and being able to take on the perspective of the female victim. Their findings further showed that the VR experience facilitated a body-swap perception among the 44 male participants but the sense of agency of being a female avatar was less strong, likely influenced by baseline psychological traits and beliefs among the men (e.g., machismo, chivalry, empathic abilities). Finally, Wu and Chen (2022) tested the influence of embodying different genders in a VR scenario in which the avatar experiences sexual harassment on implicit gender bias of the participants. Findings from the trial with 40 participants showed statistically significant effects of gender transfer among male participants, such that men were more likely to show reductions of implicit gender bias after embodiment of a female avatar.

#### VR Immersion to Promote Healing After Experiencing Sexual Violence and PTSD in the Physical World

Three articles described use of VR to deliver interventions focused on the aftermath of sexual violence and, generally, PTSD (Corno & Bouchard, 2015; Loranger & Bouchard, 2017; Loucks et al., 2019). Corno & Bouchard, (2015) present a thoughtful examination of the psychological aspects of treating PTSD in VR environments, concluding that there are two positive psychological uses of these environments: use to prevent sexual assault and use to prevent psychiatric challenges in female sexual assault survivors. Notably, these authors focus solely on VR uses among women, rather than, for example, considering the role VR might have in preventing offending behaviors among men. Loranger and Bouchard (2017) conducted what might be viewed as a controversial VR trial in which they randomly assigned 30 women (a mix of those who have and have not experienced sexual assault) to be immersed in one of two bar scenarios in which an aggressive scenario results in sexual assault, or does not. Although the authors report no adverse events in the experiment, their findings not surprisingly indicate increased and negative affect among those in the experimental condition (where assault occurred). Although plausible that exposure therapy such as this could be helpful to sexual assault survivors experiencing PTSD, more carefully conducted research appears warranted. Loucks et al. (2019) provide the only article describing use of VR to deliver exposure therapy to not only women but also men who have experienced sexual assault during their military service. Fifteen participants, of which a quarter were men, completed 12 VR exposure therapy sessions and, at 3 months after therapy, sustained positive improvements in PTSD and depressive symptoms.

## Discussion

With respect to the science and inquiry advancing our understanding, this scoping review reinforced the need for collective consensus around the definitions of terms used to describe the constructs and experiences within VR and gaming, particularly experiences of sexual violence and harassment while immersed in a gaming situation. Although the VR territory is new, many of the experiences, and their consequences, are not; this multidisciplinary body of scholars would benefit from intentional unification around working definitions, use of validated measures to assess impacts and intervention outcomes, and establishment of ethical standards and guidelines specific to VR interventions that involve sexual violence and related harms and threats as part of the simulation scenario or intervention. Increasingly, study teams are developing and testing video games to deliver interventions, and over time, it is very likely these inquiries and experiments will extend into VR environments. To ensure meaningful study findings, researchers are encouraged to collect sufficient and broad demographic data and to recruit young adults from diverse settings (e.g., beyond institutions of higher education) so that results and implications can be carefully considered for more populations and subgroups of young people.

Our scoping review and the in-depth review of gaming history to present day that accompanied the review clearly show the strength and power of the gaming industry economy, the persistence of gaming environmental characteristics that encourage harms, the short-lived remorse and remediation within the gaming industry when public outcry about harms occurs, and the unlikelihood of dramatic transformation of existing gaming and VR environments, story lines, and characters. Where there is demand, there will be supply: This economic theory truism cannot be ignored while exploring theoretical strategies to reduce harm and mitigate risks. For example, in an ideal world, companies that develop and host VR games and platforms would take proactive steps to examine gaming code and incorporate more explicit parameters that could opt a participant out of virtual sexual violence (e.g., establishing clear virtual space boundaries, instituting warnings when entering specific rooms or games). These companies could further support healthy VR experiences by establishing funding mechanisms that further our understanding of the impacts of VR harms, harassments, and incidents and advance our successes in supporting VR users who have had negative experiences or retraumatization as a result of their time spent in VR immersive environments.

Financial incentives toward nonharm in VR hold promise but are not yet widespread. In April of 2022, the European Union (EU) passed the Digital Services Act, a critical piece of legislation that requires any company providing services in the EU to monitor and rapidly remove any illegal and hateful content, including sexual content (European Commission, n.d.). The financial penalties of noncompliance are steep, in typical EU regulatory fashion, and over time, it is likely this measure will yield positive outcomes as it is tested with respect to gaming companies and VR environments.

In the United States and perhaps less obvious as a regulatory tool, the structure of Title IX has potential for impact, particularly given the status of sports video gaming as one of the best established genres within the VR entertainment sector. Recognizing that all institutions of higher education must comply with Title IX parameters, Stoever (2021) clearly describes the specific potential role that colleges (and by proxy, their nurses and other health promotion professions serving college students) could and should have in creating safe and inclusive gaming experiences. Esports is one of the fastest growing competitive markets in the world, and the gaming industry is repeating past mistakes of professional sports leagues like the National Football League, as shown through the summer 2020 outpouring of allegations of gender-based harassment, discrimination, and sexual assault from competitive gamers and streamers. Rather than waiting for a global #EToo movement to create demand for a comprehensive gender-based violence policy, colleges should affirmatively act to create responsible gaming initiatives with goals of violence prevention, player protection, and harm minimization.

Title IX is federal legislation that can and should provide an impetus to equalize access to VR and sports gaming for women. In the context of institutional policies in response to federal law, knowing the risks and potentials for unintended harm from VR is imperative. This inherently requires colleges and those working with college-age young people to have competent understanding of VR associated risks, mitigating factors, and interventions when harm occurs; educational strategies that inform everyone working with college students are imperative.

## Implications for Clinical Forensic Nursing Practice

This review underscores the absence of forensic nursing to date in the current discourse and research around sexual violence in VR environments. Nevertheless, there are bidirectional lessons to be gleaned from the current state of the science, offering guidance for clinical forensic nursing practice while also elucidating clear gaps in which forensic nursing expertise would be valuable. The first step for these possibilities is a clear awareness among forensic nurses of the problem of sexual violence, broadly, in VR and other gaming environments.

Some sexual assault nurse examiner programs have integrated basic questions in recognition of the interplay of online interactions and sexual violence (e.g., the Regions Hospital sexual assault nurse examiner program in St. Paul Minnesota includes this question series as part of history-taking and documentation, “Did patient meet assailant[s] online or through social media? If so, which?”). However, a deeper consideration not only of initial encounters via online social media but also of gaming and other VR immersive environments might be warranted in forensic nursing medical examinations and assessments. This is particularly true for those working on college campuses or with young adults and other demographics disproportionately at risk for sexual violence in VR and gaming, broadly. More explicit questions about engagement in gaming or VR could facilitate a deeper understanding of patient experiences and risk factors and, in turn, enhance understanding of the (potential) impact of this violence within certain populations of young adults, including college students.

For forensic nurses and others engaged in sexual violence prevention and health promotion efforts on college campuses, an awareness of VR-associated risks and harms can inform their supportive strategies and interventions with these healthy young adults. From direct patient care and educator/health promotion roles to roles on advisory committees and informal engagement with colleagues, explicitly including VR in discussions could show foresight and lead to earlier preventive strategies and interventions. Doing so could also solidify an important place for forensic nursing in nascent discussions at local, institutional, and national levels. Finally, the discussion of research and policy gaps and needs also serves to inform the nursing perspective and role of forensic nursing expertise in setting research priorities and policy initiatives.

## Conclusion

Through our scoping review, we see a clear overview of the state of the science, the gaps, and the opportunities centered around VR immersive environments. Across its myriad uses, VR presents critical risks and opportunities for safety and well-being that have not been fully appreciated to date by researchers, policy makers, healthcare professionals, and others. The expertise of the forensic nurse is germane and needed here, delivering trauma-informed care as trauma stewards, offering support for survivors along the continuum of needs, and developing and delivering interventions to prevent violence and to remediate offenders. From education and practice to policy making and program development, forensic nurses are perfectly positioned to promote health and mitigate harms in VR environments.

**Figure:**
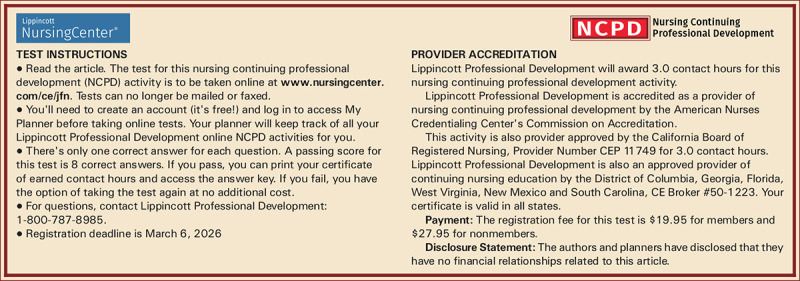


**Figure:**